# Ending Open Defecation in Rural Tanzania: Which Factors Facilitate Latrine Adoption?

**DOI:** 10.3390/ijerph110909854

**Published:** 2014-09-22

**Authors:** Stephen Sara, Jay Graham

**Affiliations:** Department of Environmental and Occupational Health, School of Public Health and Health Services, George Washington University, 2100 M St., NW Suite 200, Washington, DC 20037, USA; E-Mail: jgraham@gwu.edu

**Keywords:** sanitation, open defecation, latrine adoption, Tanzania

## Abstract

Diarrheal diseases account for 7% of deaths in children under five years of age in Tanzania. Improving sanitation is an essential step towards reducing these deaths. This secondary analysis examined rural Tanzanian households’ sanitation behaviors and attitudes in order to identify barriers and drivers to latrine adoption. The analysis was conducted using results from a cross-sectional study of 1000 households in five rural districts of Tanzania. Motivating factors, perceptions, and constraints surrounding open defecation and latrine adoption were assessed using behavioral change theory. Results showed a significant association between use of improved sanitation and satisfaction with current sanitation facility (*OR: 5.91; CI:* *2.95–11.85;* *p = 0.008*). Livestock-keeping was strongly associated with practicing open defecation (*OR: 0.22; CI* *0.063–0.75;* *p < 0.001*). Of the 93 total households that practiced open defecation, 79 (85%) were dissatisfied with the practice, 62 (67%) had plans to build a latrine and 17 (18%) had started saving for a latrine. Among households that planned to build a latrine, health was the primary reason stated (60%). The inability to pay for upgrading sanitation infrastructure was commonly reported among the households. Future efforts should consider methods to reduce costs and ease payments for households to upgrade sanitation infrastructure. Messages to increase demand for latrine adoption in rural Tanzania should integrate themes of privacy, safety, prestige and health. Findings indicate a need for lower cost sanitation options and financing strategies to increase household ability to adopt sanitation facilities.

## 1. Introduction

### 1.1. Health and Sanitation

It is estimated that poor sanitation and hygiene account for 7% of deaths in low and middle income countries (LMICs) [1]. Use of improved sanitation has been found to reduce transmission of enteric pathogens and intestinal parasites, as well as reduce morbidity and mortality, especially in children [2,3,4,5]. Open defecation (OD), which involves no method of excreta containment, increases human exposures to enteric pathogens and is considered a major risk to children’s health and development [5]. Facilitating access and use of improved sanitation is vital to reducing the transmission of diarrheal diseases [4,5]. A recent study examined the relationship between OD and child height in 65 LMICs and found that OD explained the majority of variation in child height [6]. While several observational studies have looked at improved sanitation and disease prevention, few intervention studies have explicitly focused on OD as an exposure variable, leaving limited evidence on the relationship between OD and health [7].

### 1.2. Sanitation Trends in Tanzania 

Diarrheal diseases account for 7% of deaths in children under five years of age in Tanzania [8]. Improving sanitation is an essential step towards reducing these deaths. Progress towards the Millennium Development Goals (MDG) sanitation target has been slow, especially in sub-Saharan Africa, where an estimated 25% of the population lacks access to basic sanitation [9]. The practice of OD is much more common in rural areas of sub-Saharan Africa—an estimated 35% of rural households, in contrast to 8% of urban households, report to practice OD [9].

Despite Tanzania’s high rate of urbanization (4.7%/year in 2010), 74% of the population is rural [10]. Between 1990 and 2010, the proportion of Tanzanians practicing OD increased from 8% to 12%; during this time the national population grew from 25.4 million to nearly 45 million people [9,10]. In urban Tanzania, the prevalence of OD has remained at 2% (1990–2010) while the practice has increased from 10% to 16% in rural areas [9]. As a result, an estimated 5.3 million rural inhabitants practiced OD in 2010 [10]. Given that Tanzania is estimated to be the fifth largest recipient of international aid in the water, sanitation and hygiene sector, the current trends in OD are unexpected [9].

Recent estimates based on Demographic and Health Survey data highlight considerable geographic variability in rural Tanzania’s OD prevalence and trends [11,12,13]. The practice of OD is more common in the North Central Regions and on the islands that compose Zanzibar. Half of Tanzania’s regions have seen increases in OD from 2004 to 2010, while most regions that have decreased OD prevalence have observed only modest gains (decreasing OD prevalence by <10%) [11,12,13]. In seven of the country’s 26 regions, at least 30% of the population in 2010 was estimated to practice OD [13].

### 1.3. Latrine Adoption and Behavioral Change

**Figure 1:**
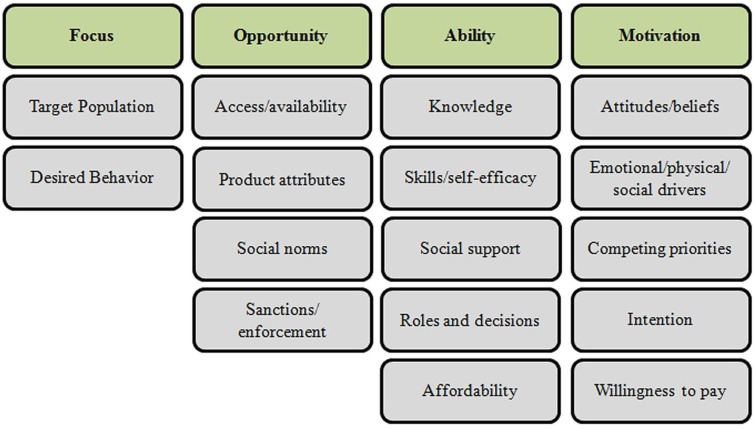
SaniFOAM Framework [14].

While several behavior change models can be applied to latrine adoption, the recently developed SaniFOAM (Focus on Opportunity, Ability, and Motivation) model was specifically developed to address sanitation and hygiene concerns. Using four categories of determinants: (1) focus; (2) opportunity; (3) ability; (4) motivation, the FOAM model is useful in identifying barriers to latrine adoption while also serving as a tool for designing, monitoring, and evaluating sanitation interventions [14,15]. Figure 1 lists relevant sanitation behavior change determinants according to the FOAM framework.

In short, if a household has the opportunity, the ability, and the motivation to adopt a behavior, behavior change will occur. If, on the other hand, a barrier arises that inhibits one of these attributes, it may lead to delayed behavior change or inaction.

In existing literature, drivers of latrine adoption tend to exist within the *opportunity* and *motivation* categories. Latrines are often attributed to an increase in cleanliness and prestige, along with improvements in safety, privacy, convenience, and social status [16,17,18,19]. Increasingly, research has documented that health benefits are usually low on the list of motivating factors for adopting latrines [17,20,21]. Common constraints to latrine adoption, on the other hand, fall primarily in the *opportunity* and *ability* categories of the FOAM model. Jenkins and Scott studied barriers to latrine access and adoption in rural Ghana and discovered that the leading constraints to building a latrine included limited space, followed by high costs, lack of construction experts, savings and credit issues and competing priorities [17]. Economic constraints, land ownership, and geographic issues are also common constraints, but are not easily affected by an individual or household [16,17,22].

Although there is strong international evidence concerning drivers and barriers to latrine adoption, little evidence is available in the context of rural Tanzania. Attention has largely been paid to the issue of upgrading “unimproved” sanitation facilities to ‘improved’ facilities, a problem that affects 77% of the country’s rural population. Given that OD is the least sanitary method of excreta disposal and the practice is becoming more common in many of Tanzania’s rural areas, this study focused specifically on identifying perceptions, barriers and drivers of latrine adoption. Results of this study can be used to inform future research and develop effective programmatic tools aimed at ending OD in rural Tanzania.

## 2. Methods

**Figure 2:**
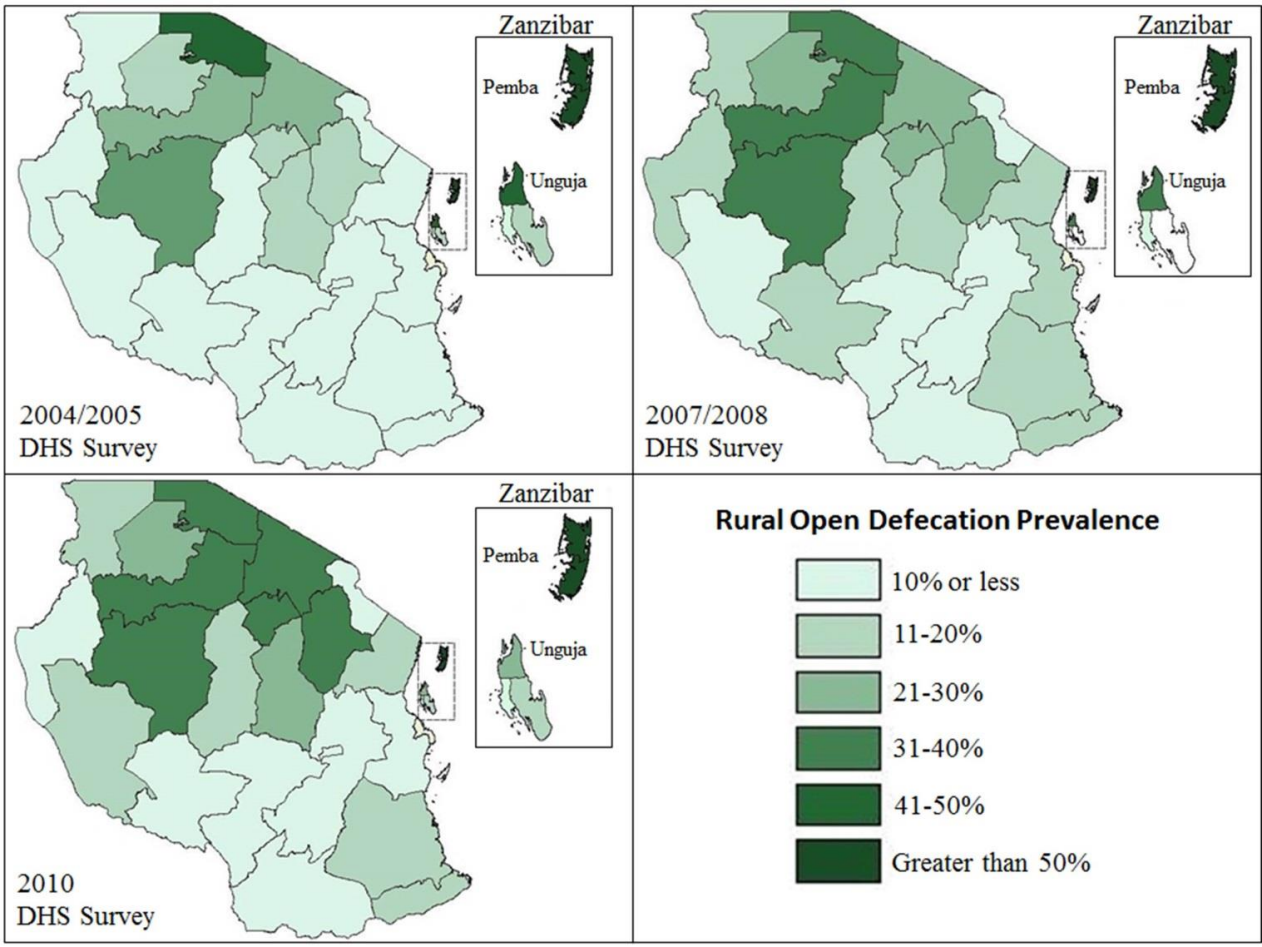
Regional Prevalence of Reported Open Defecation among Rural Households in Tanzania (2004–2010) [11,12,13].

National DHS surveys (2004, 2007 and 2010) were analyzed to provide background data on regional OD prevalence and trends in rural Tanzania. Geographic representations of DHS survey results were mapped using ArchGIS software (Figure 2).

In order to examine barriers to latrine adoption, this secondary analysis used data collected for a World Bank Water and Sanitation Program (WSP) consumer/household research study. All data used for this study are publicly available. STATA statistical software was used to test relationships between study variables. The WSP survey questions focused on perceptions, attitudes, knowledge, and habits related to OD and adopting an improved latrine [23]. Over a period of five months (July–November 2008), a total of 1000 households were surveyed in five districts of different regions: Rufiji in the region of Pwani; Iringa in the region of Iringa; Musoma in the region of Mara; Sumbawanga in the region of Rukwa; and Kiteto in the region of Manyara [23]. These districts were surveyed to represent the geographic settings of the rural population of Tanzania (coastal, central, lake, and highlands) [23]. The survey consisted of single response and multiple-response questions, as well as observational methods. Participating households were selected using multistage weighted random sampling at the ward and village level [23]. To satisfy inclusion criteria, all participating households were required to be located in rural wards within each study district. Urban and “mixed” wards were excluded from the sample [23]. Wards with less than three villages were also excluded. In total, five wards were excluded from the study. Respondents were required to be at least 18 years old and a member of the household being surveyed [23].

Pearson’s Chi Square Test of Independence and Fisher’s Exact Test were used to identify significant associations between each outcome variable and each exposure variable. The relationships between independent variables (education, district, religion, age, income and occupation, relationship to head of household) were examined. Collinearity between study variables was assessed using the variance inflation factor (VIF) test. Several multivariate logistic regression models were used to control for possible confounding factors, including; geographic location, education, occupation, religion, and age. The strongest regression models were identified through post estimation tests that determine the best fit model. Due to the low response rate on income related questions, linkstat analyses were used to compare income quintiles among the 265 respondents to income related questions. Within this sub-sample the authors examined the relationship between education, district, religion, occupation and income through multivariate regressions and VIF tests. For certain survey questions with low response rates, only exploratory data analyses were conducted.

## 3. Results

### 3.1. A Recent Trend Analysis of OD Prevalence in Tanzania

Results from a trend analysis of OD prevalence between DHS surveys conducted in 2004–2005, 2007-2008, and 2010 showed high variance in OD prevalence between Tanzania’s 26 regions. Figure 2 depicts the prevalence of OD reported by households in each of the DHS surveys. In Northern Tanzanian regions OD is becoming more prevalent, while in Southern regions OD prevalence remains relatively low. This analysis served as reference data for the secondary analysis of the WSP cross-sectional dataset used in this study.

### 3.2. Household Characteristics and Sanitation Practices

Out of the 1,000 households, 93 (9.4%) households reported practicing OD, 885 (90.3%) reported using a latrine or toilet of some type, and two households (<1%) reported the use of plastic bags, which are commonly referred to as “flying toilets”. Twenty households did not respond when asked where they currently defecate and were removed from this analysis. Table 1 shows household characteristics based on the respondent’s reported sanitation facility. Sanitation coverage varied significantly across the districts.

**Table 1:** Descriptive statistics of households practicing open defecation and households reporting latrine use.

Categoryf Characteristic	Category	Sample Description *	
		Households that report	Households reporting
		practicing OD	latrine/toilet use
		*# (%)*	*# (%)*
Respondent’s age (years)		*n = 93*	*n = 885*
	Range	18–49	18–49
	Mean	34	34
District (Region)		*n = 93*	*n = 885*
	Iringa (Iringa)	1 (1%)	237 (27%)
	Kiteto (Manyara)	33 (35%)	67 (7%)
	Musoma (Mara)	48 (52%)	179 (20%)
	Rufiji (Pwani)	11 (12%)	86 (10%)
	Sumbawanga (Rukwa)		316 (36%)
Respondent’s relationship to		*n = 93*	*n = 881*
head of household			
	Head of Household	48 (51%)	467 (53%)
	Wife	37 (40%)	331 (37%)
	Other ^†^	8 (9%)	83 (10%)
Respondent’s level of		*n = 93*	*n = 871*
education attended ^‡^	None	28 (30%)	66 (8%)
	Primary	59(64%)	724 (83%)
	Secondary	6 (6%)	79 (9%)
	Higher		2 (<1%)
Houshold weekly income (USD) ^§^		*n = 36*	*n = 229*
	<$5	7 (20%)	17 (7%)
	$5–$9	12 (33%)	59 (26%)
	$10–$17	6 (17%)	60 (26%)
	$17–$26	4 (11%)	26 (11%)
	$26–$34	3 (8%)	27 (12%)
	>$34	4 (11%)	40 (18%)
Primary occupation (hh?)		*n = 93*	*n = 881*
	Works on own farm	65 (70%)	728 (83%)
	Works for pay	3 (3%)	62 (7%)
	Unpaid in family business	1 (1%)	25 (3%)
	Unpaid worker on family farm	3 (3%)	27 (3%)
	Livestock-keeping	15 (16%)	2 (<1%)
	Fisherman	5 (6%)	9 (1%)
	Other ^||^	1 (1%)	27 (3%)

Notes: * Sample size changes due to the option provided each respondent to refrain from answering each question. **^†^** “Other” includes the following responses; mother-in-law, grandmother, father, father-in-law, grandfather, son, daughter. **^‡^** Level of education is level attended, not necessarily graduated. **^§^** USD estimates are based upon exchange rate for 30 September 2008 (midpoint of this study)—$1 = 1165 Tsh. **^||^** “Other” includes the following responses; receives money from spouse/other, homestead member, unemployed.

### 3.3. Identifying Geographic and Socio-Demographic Factors Associated with the Practice of Open Defecation

As displayed in the Table 2, several characteristics were significantly associated with defecation status after controlling for confounding. The authors tested for collinearity among variables using the VIF test, which resulted in no collinearity among independent variables. Given the limited number of households reporting to practice OD in Sumbawanga district and Iringa district, those two districts were not included in the statistical model analyzing the association of district and OD (Table 1). Although the WSP survey was only conducted in one district of each region (typically regions have 5–10 districts), these results were fairly consistent with DHS region survey results, showing very low OD prevalence in Sumbawanga and Iringa districts (cite DHS). The prevalence of OD reported in the remaining three survey districts (Rufuji, Musoma, and Kiteto) also reported similar levels of OD when compared to DHS region survey results. Respondents from the Rufiji district had a 2.99 greater odds (*CI: 1.25–3.33*) of reporting to use a latrine/toilet facility when compared to respondents from Kiteto district, for example. This WSP study sample supported the background DHS data analysis, and indicated that there was a substantial difference in rural household toilet use between geographic areas.

**Table 2:** Bivariate and multivariate models of association between descriptive characteristics and open defecation *.

Characteristics		Bivariate				Multivariate		
		*OR*	*95% CI* ^†^	*p-value*		*OR*	*95% CI*	*p-value*
*District*	Kiteto	ref ^‡^				ref		
	Musoma	1.84	1.09–3.10	0.023				n.s. ^§^
	Rufiji	3.85	1.81–8.18	<0.001		2.99	1.36–6.57	n.s.
*Attended*	No							
*School*	Yes	5.26	3.16–8.75	<0.001		2.32	1.25–4.33	0.008
*Occupation*	Works on own farm	ref				ref		
	Works for pay			n.s.				n.s.
	Receives income from spouse			n.s.				n.s.
	Homestead member			n.s.				n.s.
	Unpaid in family business			n.s.				n.s.
	Unpaid on family farm			n.s.				n.s.
	Unemployed			n.s.				n.s.
	Livestock-keeping	0.11	0.002–0.05	<0.001		0.22	0.063–0.75	0.016
	Fisherman			n.s.				n.s.
*Religion*	Traditional/Pagan	ref				ref		
	Muslim/Christian	7.03	3.06–16.15	<0.001		4.03	1.69–9.66	0.002

Notes: * The outcome variable was dichotomous (0 = Open defecation, 1 = latrine/toilet use). **^†^** “CI” means Confidence interval”. **^‡^** “ref” refers to the referent. **^§^** “n.s.” means not significant.

Education was significantly associated with latrine or toilet use. While only 8% of households using a latrine or toilet facility had never attended school, nearly one-third (30%) of households practicing OD had never attended school (Table 1). Those respondents who reported having attended school had 5.26 greater odds (*CI: 1.25–4.33*) of using a latrine/toilet facility when compared to respondents who reported never attending school. Further analysis showed no significant association between the number of years of education and latrine/toilet use.

Certain occupations, namely livestock-keeping, were significantly associated with OD. Among households practicing OD, 15 (16%) earned the majority of their income from livestock-keeping, compared to 2 (<1%) respondents using a latrine or toilet facility (Table 1). Those respondents who reported earning the majority of their income from livestock-keeping had 0.11 odds (*CI: 0.063–0.75*) of using a latrine/toilet facility when compared to respondents who worked on their own farm. No other occupation had a significant association with latrine/toilet use. Religion also presented a statistically significant association with defecation practice. Table 2 shows that those respondents who reported practicing Christianity or Islam had 4.03 greater odds (*CI: 1.69–9.66*) of using a latrine/toilet facility compared to respondents who reported practicing Traditional African or Pagan religions.

Other descriptive characteristics, such as age and position in the household were also analyzed with no significant associations to report. Income was not included in the regression model of Table 2 due to a low response rate. Likelihood ratio tests were used to explore the relationship between household income and defecation practices among the 265 respondents who responded to the income questions. Although this sample was substantially lower than the survey sample and response bias could not be accounted for, the results of this exploratory test yielded no significant relationship between income and reported defecation practices. While other factors, such as education, can sometimes be used as proxy indicators for income or SES, a variance inflation factor test revealed no collinear relationship, prohibiting the use of education as a proxy indicator for income [24].

### 3.4. Associations between Defecation Practices and Satisfaction 

A similar multivariate regression was conducted to determine which factors were associated with household satisfaction with current sanitation facility, displayed in Table 3. A significant association was discovered between a household’s defecation practices and it’s satisfaction with the current household sanitation facility. The majority of households practicing OD (85%) were dissatisfied with their current place of defecation, while just over half (55%) of households using latrines or toilet facilities were dissatisfied. Respondents reporting latrine or toilet use had 2.05 greater odds (*CI: 2.95–11.85*) of reporting satisfaction with their current place of defecation compared to respondents who reported practicing OD. Common reasons for dissatisfaction among latrine users were related to superstructure and flooring issues. Safety and cleanliness were the leading reasons for dissatisfaction among households practicing OD, and were also concerns for households using latrine/toilet facilities. Interestingly, no statistical associations were identified between satisfaction in defecation practices and education, district, occupation, religion, position in the household, or age of the respondent.

Households reporting to practice OD were compared based on their intention, or no intention, to construct a latrine. Table 3 shows that both groups had similar perceptions of latrine access and use. The majority of respondents practicing OD agreed that it is important to improve latrines to stay healthy. When asked to list the reasons for owning a latrine, the two most common answers listed by respondents in both groups were good health and avoiding the contamination of the environment. The top four characteristics of a good latrine reported among households practicing OD were: (1) having a “good” superstructure/floor; (2) a clean latrine; (3) no odor, and (4) the presence of disinfectants and water. Respondents that reported practicing OD also largely agreed that families cannot expect help from others to upgrade sanitation facilities. Most households noted that it is difficult to access information on how to construct a latrine and that community organizations are not available to assist in latrine construction. Good health was the most important reason to own a latrine among households planning to build a latrine (60%), while avoiding environmental contamination was the most important reason among households without plans (54%).

### 3.5. Comparing Household Perceptions Based on Plans to Construct a Latrine

Ninety households reporting to engage in OD responded to the question, “Do you have plans to construct a latrine”? The analysis in Table 3 compared households that reported having plans to build a latrine (69%) and those that did not have plans (31%).

**Table 3:** Comparison of household perceptions based on plans to construct latrine among households reporting to practice OD *.

Survey Question			Response Category	Have Plans to Construct Latrine ^†^ # (%)	
				Yes	No
				62 (69%)	28 (31%)
				*n = 62*	*n = 28*
*Satisfied with current place of defecation (single response)*			Satisfied/very satisfied	3 (5%)	4 (15%)
			Neutral	2 (3%)	2 (7%)
			Unsatisfied/very unsatisfied	57 (92%)	22 (78%)
				*n = 124*	*n = 61*
*Reasons for dissatisfaction with current place of defecation (open ended question, up to 3 responses)*			Unsafe	49 (40%)	24 (39%)
			Unclean	30 (24%)	13 (21%)
			Temporary	17 (14%)	3 (5%)
			Bad odors	7 (6%)	6 (10%)
			Poor condition	5 (4%)	5 (8%)
			Share with others	2 (2%)	2 (3%)
			Construction concerns ^‡^	4 (3%)	3 (5%)
			Other ^§ ^	10 (8%)	5 (8%)
				*n = 60*	*n = 26*
*Main reason to have latrine (open ended question, single response)*			Good health/avoid disease	36 (60%)	5 (19%)
			Avoid contaminating the environment	16 (27%)	14 (54%)
			Every household must have a latrine	2 (3%)	5 (19%)
			Other ^||^	6 (10%)	2 (8%)
				*n = 171*	*n = 77*
*Characteristics of good latrine (open ended question, up to 3 responses)*			Superstructure/floor ^¶^	92 (54%)	34 (44%)
			Clean	27 (16%)	12 (16%)
			No odors	13 (8%)	7 (9%)
			Disinfectants & water available for cleaning hands/anus	10 (6%)	10 (13%)
			Permanent	12 (7%)	5 (6%)
			Easy to clean	7 (4%)	2 (3%)
			Not full	6 (4%)	1 (1%)
			Ceramic pan	3 (1%)	1 (1%)
			Other **	1 (1%)	5 (7%)
				*n = 62*	*n = 28*
*Families can’t expect help from others to upgrade sanitation facility*			Disagree/strongly disagree	22 (35%)	9 (32%)
			Agree/strongly agree	40 (65%)	19 (68%)
				*n = 62*	*n = 28*
*Easy to access info. on how to construct latrine*			Disagree/strongly disagree	41(66%)	25 (89%)
			Agree/strongly agree	21 (34%)	3 (11%)
				*n = NA*	*n = 25*
*For those not planning to build, why? (open question, single response)*			Financial constraints	NA	11 (44%)
			Tradition, cultural beliefs	NA	6 (24%)
			Can’t find materials	NA	3 (12%)
			Other ^††^	NA	5 (20%)
*Single greatest problem to *				*n = 62*	*n = 27*
*household sanitation in this *			Lack of clean water	14 (23%)	16 (59%)
*community*			Open defecation	13 (21%)	5 (19%)
			Poor latrines	14 (23%)	2 (7%)
			Collapsing latrines	9 (14%)	3 (11%)
			Other ^§§^	12 (19%)	1 (4%)
				*n = 62*	*n = 28*
*OD is normal in community*			Disagree/strongly disagree	21 (34%)	3 (11%)
			Agree/strongly agree	41 (66%)	25 (89%)
				*n = 62*	*n = 28*
*People who defecate in open put entire community at risk of disease*			Strongly disagree	1 (1%)	1 (4%)
			Disagree/strongly disagree	8 (13%)	1 (4%)
			Agree/strongly agree	52 (84%)	25 (89%)
			Don’t know	2 (3%)	1 (4%)
				*n = 62*	*n = 28*
*Continued open defecation will put the whole community at risk of getting sick*			Disagree/strongly disagree	8 (13%)	1 (4%)
			Agree/strongly disagree	53 (84%)	26 (93%)
			Don’t know	1 (2%)	1 (4%)
				*n = 62*	*n = 27*
*People are worried about children getting sick from poor sanitation*			Disagree/strongly disagree	9 (15%)	4 (15%)
			Agree/strongly agree	52 (85%)	23 (85%)

Notes: * Throughout the table each question lists a different sample size based on how many respondents answered the question, as participants were allowed to opt out of answering each question. Additionally, for some questions participants were allowed to answer with multiple responses, resulting in sample sizes larger than the number of respondents. **^†^** Three respondents practicing OD were not included in this table due to non-response. **^‡^** “Construction concerns” includes; no roof, no door, not modern, unstable, no superstructure, leaking. **^§^** “Other” includes; no privacy, too many flies/mosquitoes, dark, no place to wash hands, dislike everything, nothing wrong. **^||^** “Other” includes; privacy, avoid embarrassment, convenience. **^¶^** Superstructure and floor materials were reported in multiple ways, but were categorized as one variable. ** “Other” includes; lined pit, pit cover, not shared. **^††^** “Other” includes; poor soil, land lord’s responsibility, unaware of advantages. **^§§^** USD estimates are based upon exchange rate for 30 September 2008 (midpoint of this study)—$1 = 1165 Tsh.

Given the small sample size, only bivariate and/or exploratory analyses were conducted. Only 46% of households without plans to build a latrine had attended primary school, while 87% of those with plans had attended primary school; an association that was statistically significant (*p-value < 0.001*). Over half (53%) of households without plans to build a latrine earned the majority of their income from keeping livestock, while no households planning to build a latrine earned the majority of their income from livestock-keeping (*p-value < 0.001*)*.*

### 3.6. Perceptions of Open Defecation 

Many similarities were observed among households practicing OD, regardless of their future plans to build a latrine (displayed in Table 3). The majority of respondents practicing OD perceived the practice as normal within their communities. As previously mentioned, the majority of respondents practicing OD (85%) were dissatisfied with their current place of defecation. The top two reasons for dissatisfaction among households that practice OD were safety concerns and unclean conditions. Households that practice OD largely agreed that the practice puts the entire community at risk of illness, and respondents were concerned about children becoming ill due to poor community sanitation.

### 3.7. Drivers and Barriers to Latrine Construction among Households Planning to Build

Table 4 contains responses from households practicing OD but who are planning to build a latrine. The majority of households that planned to build a toilet (93%) chose to build some version of a pit latrine—latrine with no slab (75%), latrine with slab (11%), or ventilated pit latrine (7%)—because they were affordable and easy to construct. Among these households, the most common benefits to owning a latrine were considered privacy (57%), increased safety (17%), and respect from neighbors or increased social status (14%). Interestingly, these responses differed from responses to a similar question in Table 2, where good health and environmental contamination were the main reasons reported for having a latrine.

**Table 4:** Description of households practicing open defecation who are planning to build a basic sanitation facility *.

Survey Question	Response Category	# (%)
		*n = 108*
*Main reasons for selecting toilet type* ^†^ *(open ended question, 3 responses allowed)*	Easy to construct	37 (34%)
	Affordable	35 (32%)
	Durable	8 (7%)
	Easy to clean	7 (6%)
	Children can use	6 (5%)
	No smell	4 (4%)
	Don’t know	1 (<1%)
	Other ^‡^	10 (9%)
		*n = 56*
*Main benefit to having new latrine (open ended question, single response)*	Privacy	32 (57%)
	Increased Safety	10 (18%)
	Neighborly respect/increased status	8 (14%)
	Other ^§^	6 (11%)
		*n = 55*
*Planned method of payment (single response)*	Save money	42 (76%)
	Sell livestock or agriculture products	5 (9%)
	Borrow money from friends	3 (5%)
	No casual labor	1 (2%)
	Don’t know	4 (7%)
		*n = 37*
*Perceived length of time to save sufficient funds for latrine construction*	≤2 weeks	2 (5%)
	2 weeks to 1 month	4 (11%)
	1–3 months	12 (32%)
	>3 months	19 (51%)
		*n = 42*
*Started to save money for new latrine construction?*	Yes	16 (38%)
	No	26 (62%)
		*n = 109*
*Where will you get information on improving latrine (all that apply)*	Local government	27 (25%)
	Friends/Neighbors	25 (23%)
	Mason/Fundi/Service Provider	23 (21%)
	Health Committee members	14 (13%)
	Environmental Health officer	6 (6%)
	Radio	5 (4%)
	Landlord	4 (4%)
	Other ^||^	5 (4%)
		*n = 133*
*Main constraint to building latrine (open question, up to 3 responses)*	Cost of the latrine	61(46%)
	Lack of ability to save or access credit	28 (21%)
	Accessing the necessary building materials	25(19%)
	No one to build/advise	7 (5%)
	Land type/lack of space	6 (5%)
	Water table/soil conditions	3 (2%)
	Don’t know/nothing	3 (2%)

Notes: * Throughout the table each question lists a different sample size based on how many respondents answered the question, as participants were allowed to opt out of answering each question. Additionally, for some questions participants were allowed to answer with multiple responses, resulting in sample sizes larger than the number of respondents. **^†^** Four respondents planning to build sanitation facilities were excluded from this table because the authors concentrated only on basic facilities (latrines w/slab, latrine w/o slab, ventilated latrine). **^‡^** “Other” includes; easy to improve, modern, familiar type, limited materials, don’t see feces. **^§^** “Other” includes; modern latrine, health reasons, odorless latrine, and environmental reasons. **^||^** “Other” includes; myself/ourselves, technicians, service providers that pass through, don’t know.

Leading constraints among households planning to build a latrine were latrine cost (46%), inability to save or access credit (21%) and no one to build/advise the household on latrine construction (19%). Leading constraints among households with no plans to build a latrine were financial constraints (44%) and traditional or cultural beliefs regarding defecation practices (24%).

## 4. Discussion

### 4.1. Focusing Sanitation Efforts

The analysis revealed that household sanitation practices differed by geographic region, education level, religion, and among households engaged in certain occupations. Similar to estimates from DHS surveys, significant differences in OD prevalence among districts were found in this analysis. The prevalence of OD among rural households varied from 0% in Sumbawanga District to 33% in Kiteto District. DHS survey trends indicated that most regions are either experiencing an increase in OD prevalence, or they are maintaining a high OD prevalence.

Education has often been found to be associated with improvements in sanitation and hygiene at the household level [5]. This analysis suggests that education may have been an important factor in defecation practice and intention to build a latrine/toilet facility among rural households in Tanzania. Not only was there a statistically significant difference in education between households practicing OD and those using a latrine or toilet facility, but it appears that differences also existed within the OD population. Primary education attendance was much higher among households planning to build a latrine (87%) compared to households that were not planning to build (36%), indicating that education will may have positive spillover effects on defecation attitudes and behaviors.

The significant relationship between livestock-keeping and OD suggests a possible target population for future studies and interventions. Although the sample size was too low for in depth statistical analysis, the relationship between livestock-keeping and intention to build a latrine warrants more robust studies on this topic. To the knowledge of the authors, only one previous study found an association between cattle ownership and sanitation-related practices [19]. Perhaps households that earn their income from livestock-keeping are forced to travel significant distances while tending to their herds, resulting in a lack of access and a lack of *opportunity* to use sanitation facilities. It is also possible that after witnessing livestock open defecate on a daily basis, livestock-keepers assume there is little or no additional risk if they practice OD themselves. These novel findings should be explored in future studies in order to better understand sanitation perceptions and motivations among livestock keepers.

Interestingly, livestock keepers reported making varying amounts of money from their business. Despite a low response-rate among livestock keepers to income related questions [5], four different income levels were reported. Despite the variance in income level, 0 of the 5 respondents had ever attended school. Although this finding is includes a very small sample size, it may suggest that sanitation behaviors among livestock keepers are not associated with income level, but rather by social factors.

The topic of excreta reuse would be interesting to explore in this context. While reuse of human and animal excreta is not generally an income generator for rural households in LMICs, there are benefits to the reuse of excreta. The safe reuse of excreta is known to increase agricultural productivity by rejuvenating nutrients in the soil [25]. This practice has the potential to positively impact live-stock keepers by decreasing environmental exposures to harmful pathogens and by increasing the productivity of pasture lands. Although safe excreta reuse may be a viable option, the process of creating safe fertilizer from excreta must be done correctly. If the excreta does not undergo the proper decomposition process, it has the potential to increase human environmental exposures to pathogens. When programmers weight the viability of this intervention, education and behavior change activities must be closely monitored to ensure the success of the program.

### 4.2. Increasing Opportunity and Motivation

Households reporting to practice OD were overwhelmingly unsatisfied with the practice due to perceptions of it being unsafe and unclean. While dissatisfaction of open defecation primarily involved concerns of personal well-being, the perceived benefits of latrine adoption were a mix of personal well-being and social motivations. Households with intentions to build a latrine appeared to place a stronger connection between health benefits and latrine adoption than households that had no intention to build a latrine. In addition to the broad concerns of health and environmental contamination, respondents were driven towards latrine adoption by aspirations of privacy, safety, and social status. These findings present the need for in depth exploration around possible contributing factors such as education level, occupation, religion and other social factors.

Future interventions and behavior change communication campaigns can influence household attitudes and subjective norms towards sanitation behaviors in rural Tanzania. Initiatives that promote household desires for improvements in privacy and safety will likely influence these attitudes. While this analysis suggested health as a possible driver of latrine adoption, the relationship between health and sanitation improvements in rural Tanzania should be explored further. Messages concerning social status and community health may assist in redefining social norms within rural communities. Respondents overwhelmingly valued “good” superstructure and flooring as characteristics of a good latrine. It is suggested that future interventions correlate improvements in *opportunity* determinants with the *motivational* determinants of increased privacy, safety, prestige and possibly with health improvements.

### 4.3. Increasing Ability

Although behavior change communication campaigns will assist in increasing opportunities and motivation for latrines among rural households, the larger issue appears to revolve around financial barriers and a household’s ability to adopt a latrine. Of the 28 households without intentions to build a latrine, nearly half listed financial constraints as the most influential factor in the their decision. These results are consistent with previous studies on latrine adoption [17,21]. If constraints are perceived as unchangeable to household members, they result in an end to planning latrine adoption at an early stage of behavior change models [26]. Common constraints to moving from intention to latrine adoption also revolved around financial constraints, such as; the cost of the latrine, the lack of ability to save, and no access to credit. Due largely to these financial constraints, only 13 of 62 households (21%) with the intention to build a latrine believed that they would own a latrine in one year’s time. Accessing the necessary building materials and construction information were also common barriers. Thus, studies and programs are needed that explore how to address a household’s ability to change their sanitation behaviors. Increasing access to lower cost sanitation facilities or building materials and augmenting opportunities to save money or access credit are vital components for future sanitation improvement interventions within the rural Tanzanian context.

## 5. Limitations and Conclusions 

The study is only generalizable to the rural populations of specific Tanzanian districts and does not include urban and peri-urban populations. The sample size of this study was relatively small, especially when conducting sub-analyses among households reporting to practice OD. While results provide insight into factors associated with intention to adopt latrines, no follow-up was conducted to determine if households with intent to adopt latrines actually followed through with construction and use. Furthermore, this study does not analyze the effects of broader factors such as policies, economic factors, or macro-social influences (*i.e.*, ethnicity, religion) on latrine access and use.
